# Repeated Oral Administration of Ivermectin Belatedly Induces Toxicity and Disrupts the Locomotion and Neuropsychiatric Behavior in Rats

**DOI:** 10.1021/acsomega.4c09536

**Published:** 2025-03-28

**Authors:** Antônio Alvenir Comis-Neto, Natália Silva Jardim, Caroline Brandão Quines, Matheus Chimelo Bianchini, Jacqueline Gomes, Weslei Talhaferro Batista, Daiana Silva de Ávila, Sandra Elisa Haas, Suzan Gonçalves Rosa, Simone Pinton

**Affiliations:** a Federal University of Pampa, Campus Uruguaiana, Uruguaiana, Rio Grande do Sul 97508000, Brazil; b Department of Biomedicine, Regional University of the Northwest of the State of Rio Grande do Sul (UNIJUÍ), Campus Ijuí, Ijuí,98700-000Rio Grande do Sul ,Brazil; c Department of Biochemistry, Federal University of South Fronteira, Campus Chapecó, Chapecó,89815-899Santa Catarina ,Brazil

## Abstract

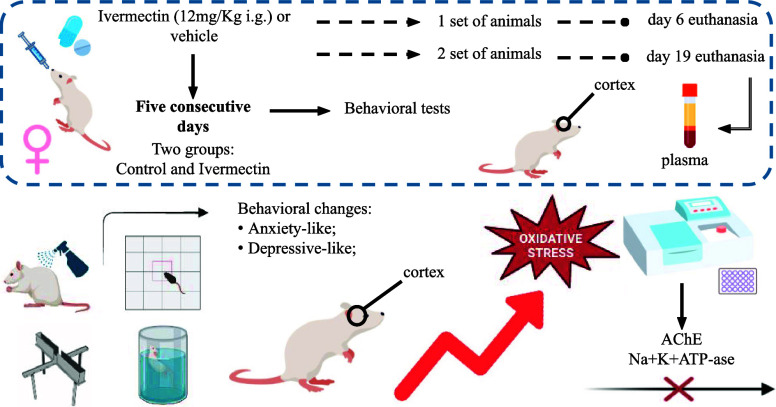

In 2020, the World Health Organization declared that COVID-19, caused by the SARS-CoV-2 virus, is a pandemic. This led to severe respiratory syndromes and overwhelmed hospital capacities alongside the widespread, yet unproven, use of drugs like ivermectin. Amidst growing concerns over the consequences of frequent ivermectin use, this study aims to examine its toxicological effects following repeated dosage in rats. Female Wistar rats received a daily dose of 12 mg/kg of ivermectin intragastrically for 5 days. Two groups were studied: one euthanized 24 h post the final dose (early protocol) and the other 14 days later (late protocol). The rats underwent tests for locomotion and anxiety- and depression-like behaviors. Additionally, blood and cortex samples were analyzed for acetylcholinesterase and Na+/K+-ATPase activities, oxidative stress levels, and liver and kidney function markers. The early protocol results showed decreased locomotion and increased signs of anxiety and depression in the rats, along with Na+/K+-ATPase inhibition and oxidative stress. In the late protocol, signs of persistent depression-like behavior and hyperlocomotion were observed, coupled with heightened oxidative stress, as indicated by increased reactive oxygen species and disrupted catalase activity. Moreover, the dual inhibition of acetylcholinesterase and Na+/K+-ATPase activities seems to underlie the behavioral alterations seen in the late protocol. The study also noted ivermectin’s potential hepatotoxic effects, corroborating previous findings of elevated liver enzyme levels and severe drug-induced liver injury cases, as well as delayed neuropsychiatric and behavioral changes.

## 1 Introduction

Since its designation as a Public Health Emergency of International Concern by the World Health Organization in January 2020, the COVID-19 pandemic has led to more than 7 million deaths worldwide by March 2024.^1^ In response to the lack of specific treatments, efforts have focused on repurposing existing medications to fight the disease. Ivermectin, an antiparasitic drug discovered in 1973.^2^ Despite controversies surrounding its use, ivermectin garnered attention as a potential therapy for COVID-19, as it was supported by an *in vitro* study demonstrating its efficacy in reducing the level of replication of SARS-CoV-2.^3^

Ivermectin is officially approved for treating parasitic infestations in humans, such as lice and worms, and is also used in veterinary medicine. However, its efficacy against COVID-19 has not been definitively proven.^4^ Despite this, widespread self-medication with ivermectin has occurred, driven by misinformation and, at times, government endorsements, leading people to use it in varying doses for COVID-19 treatment or prevention.^5,6^

One study during the pandemic found that a 5 day course of ivermectin at a dose of 12 mg was safe and led to quicker virologic reduction in COVID-19.^7^ Conversely, another study with outpatients showing mild to moderate symptoms, treated with up to 600 μg/kg daily for 6 days, showed no significant improvement compared to a placebo, suggesting ivermectin’s ineffectiveness in these cases.^8^ Thus, the data on ivermectin’s efficacy against COVID-19 are mixed, with some studies finding unreliable results.^9^

While generally safe, indiscriminate use of ivermectin has sparked concerns over potential adverse effects due to insufficient toxicological data. Research has reported severe neurological adverse effects, including pruritus, headache, dizziness, inability to walk, confusion, loss of consciousness, seizure, encephalopathy, coma, and tremors.^10^ Moreover, evidence has shown that ivermectin toxicity predominantly affected older male patients who consumed in individuals, particularly older men taking higher-than-recommended doses or using veterinary formulations.^11^ Chronic and acute toxicity has been observed, with symptoms ranging from neuropsychiatric to gastrointestinal and musculoskeletal issues.^11^

Evidence has also shown that rats exposed to repeated doses of ivermectin exhibited elevated levels of liver enzymes, indicating potential hepatotoxicity.^12,13^ Furthermore, exposure to emamectin benzoate, an insecticide within the avermectin family and possessing a molecular structure similar to that of ivermectin, adversely affects various aspects of brain function. These include motor behavior, coordination, and cognitive abilities.^14^ The indiscriminate use of high doses underscores the need to further explore the neurobehavioral effects of ivermectin and its potential clinical implications. Therefore, this study aims to assess the potential toxicological effects of ivermectin on animal behavior, specifically regarding depressive and anxiety-like behaviors, using high doses. It also seeks to analyze the mechanisms underlying these effects in rats.

## 2 Materials and Methods

### 2.1 Animals

Adult female Wistar rats (60 days old, representing 10% of their lifespan; weight 170–250 g) were obtained from a local breeding colony. The animals were housed in cages with *ad libitum* access to food and water. They were maintained in a separate room with air conditioning at 22 ± 2 °C, following a 12 h light/12 h dark cycle with lights on at 7:00 a.m. Animal care and all experimental procedures were conducted in accordance with the National Institute of Health Guide for the Care and Use of Laboratory Animals^15^ and were approved by the Experimental Animal Ethics and Use Committee at the Federal University of Pampa, Brazil (CEUA no. 003/2022). All efforts were made to minimize the number of animals used and to alleviate their suffering.

### 2.2 Experimental Protocol

The 80 rats were divided into two groups: a control group and a treatment group receiving ivermectin. Two distinct sets of 40 animals (10 per group) were used: one for behavioral data and the other for tissue testing. Each group received a daily dose of either ivermectin (12 mg/kg) or a placebo (distilled water) via gavage over a 5 day period (from day 1 through day 5). The 5 day treatment duration was based on Ahmed et al., who tested the viral clearance and safety of ivermectin among adult SARS-CoV-2 patients.^7^ The dose of 12 mg/kg was selected to assess the toxicological effects of ivermectin at a high dose, above the standard therapeutic dose. This dose corresponds to approximately 1/4 of the oral LD50 for rats, which is reported to be 51.5 mg/kg.^16^ This high dose was chosen because it exceeds the typical pharmacological dose and allows for the evaluation of potential toxicological effects, particularly regarding depressive and anxiety-like behaviors. The rats were euthanized either 24 h after the last dose (on day 6) or 14 days after the final dose (on day 19) to evaluate both acute and late-stage toxicological effects. Behavior assessments were conducted on days 4 and 5 to minimize the acute impact of ivermectin treatment while monitoring behavioral changes.

**Figure 1:**
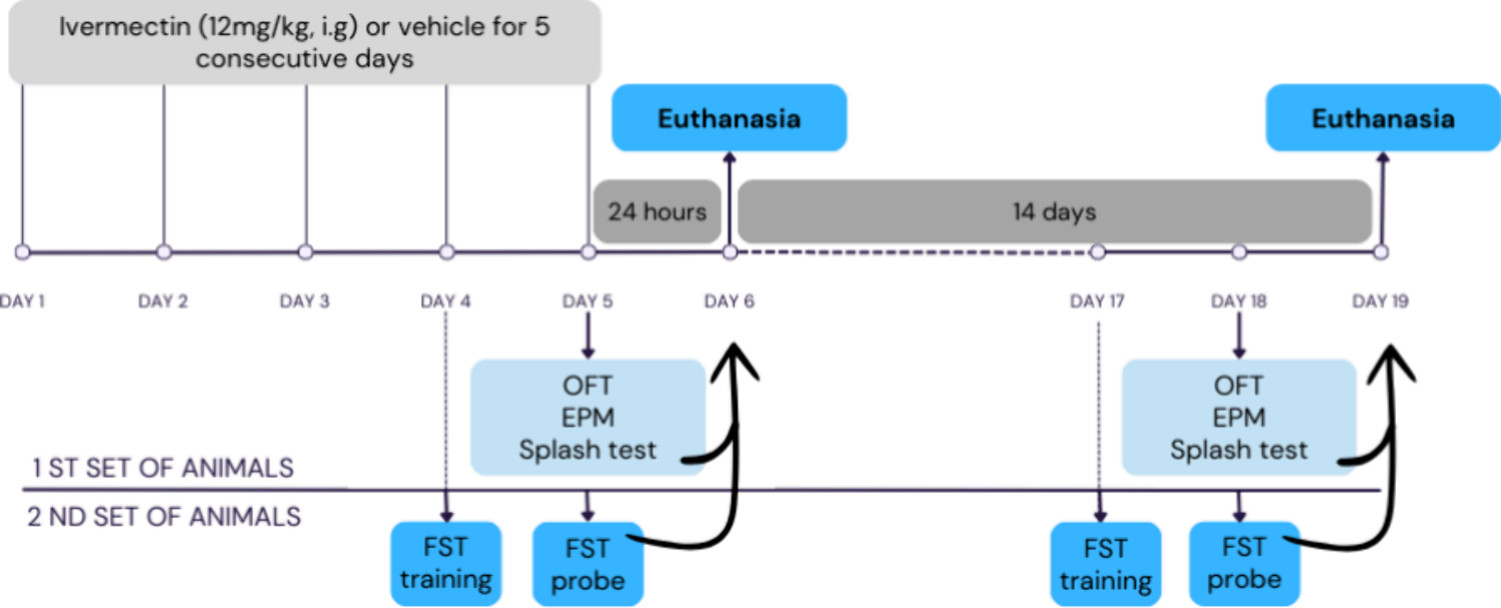
Experimental design of the early and late protocols.

Two sets of rats were evaluated using different protocols for early and late behavior (Figure 1): the first set underwent the open field test (OFT), elevated plus maze (EPM), and splash test on either days 5 or 18. The second set was assessed for depressive-like behaviors, undergoing forced swim tests (FST) on days 4 or 17, followed by a probe test on the subsequent day (days 5 or 18, respectively). The researchers monitored general health indicators throughout the study such as fur condition, tremors, lethargy, diarrhea, and body weight changes. Following the experimental period, rats were euthanized by decapitation on days 6 or 19, blood samples were collected with ethylenediamine tetraacetic acid (EDTA) as an anticoagulant, and the prefrontal cortex tissues were harvested for further analysis.

Rats were exposed to ivermectin (12 mg/kg) or a vehicle for five consecutive days and underwent various behavioral tests during the treatment period (early protocol) or in the days following exposure (late protocol). Subsequently, rats were euthanized either 24 h or 14 days after the last administration of ivermectin.

### 2.3 Behavioral Tests

#### 2.3.1 Open Field Test

The OFT was conducted using an apparatus composed of a 40 × 40 cm plywood arena with 50 cm high walls 50 cm high. The arena floor was divided into 16 equal squares by black lines (4 rows × 4 columns). Animals were placed in the center of the arena to freely explore the open field for 5 min. The number of crossings and rearings were recorded.^17^

#### 2.3.2 Elevated Plus Maze

The maze consists of a wooden structure 50 cm above the ground, featuring two open opposite arms (50 cm long × 10 cm wide) intersecting with two closed arms of the same dimensions but with walls 40 cm high. Initially, animals were placed at the intersection of the maze.^18^ They were allowed to explore the maze for 5 min. During this period, the animals’ behaviors were recorded: the number of entries and total time spent in the open arms in seconds.

#### 2.3.3 Splash Test

The splash test involves squirting a 10% sucrose solution over the dorsal coat of a rat. The sucrose solution, due to its viscosity, soils the rats’ fur. Subsequently, anhedonic behavior is assessed by measuring the duration of grooming behavior for 5 min as an indicator of self-care and motivational behavior.^19^

#### 2.3.4 Forced Swim Test

The FST was performed with minor modifications to the original method.^20^ Rats were individually placed in open plastic cylinders (40 cm high, 30 cm in diameter) containing 25 cm of water at 25 ± 1 °C. The rats were allowed to swim for 15 min before being returned to their cages. In the test session, conducted 24 h later, the rats underwent the FST again, with immobility time measured for 5 min. A rat was considered immobile when it floated motionless in water, making only movements necessary to keep its head above water. Data were reported as immobility time in seconds.

### 2.4 *In Vivo* and *Ex Vivo* Assays

#### 2.4.1 Acetylcholinesterase Activity

Prefrontal cortex samples were homogenized in a 0.25 M sucrose buffer (1/10, weight/volume) and centrifuged at 900 × *g* at 4 °C for 15 min. Acetylcholinesterase (AChE) activity was measured by spectrophotometry at 412 nm and expressed in micromoles of acetylcholine per hour per milligram of protein.^21^

#### 2.4.2 Oxidative Stress Parameters

Homogenates of prefrontal cortex samples were prepared in 0.05 M Tris/HCl buffer (pH 7.4) (1/10, w/v). The homogenate was centrifuged, and the supernatant (S1) was used for oxidative stress and Na^+^/K^+^-ATPase activity assays.

##### 2.4.2.1 Reactive Oxygen Species Levels

To assess the production levels of reactive oxygen species (ROS) in tissue homogenate, a 10 μL aliquot of S1 was incubated with 10 μL of 2′,7′-dichlorofluorescein diacetate (DCHF-DA; 1 mM). The ROS levels were determined by using spectrophotometry. This method measures the oxidation of DCHF-DA to fluorescent dichlorofluorescein (DCF), which indicates intracellular ROS detection. The intensity of DCF fluorescence emission was recorded at 520 nm (with excitation at 480 nm) 1 h after adding DCHF-DA to the medium. ROS levels were expressed in fluorescence.^22^

##### 2.4.2.2 Catalase Activity

The enzymatic reaction commenced with the addition of a 20 μL aliquot of S1 and substrate (H_2_O_2_, 0.3 mM) to a medium containing 50 mM phosphate buffer (pH 7.0) and was measured at 240 nm. Catalase activity was quantified in units (one unit decomposes 1 μmol of H_2_O_2_ per minute at pH 7 and 25 °C) per milligram of protein.^23^

#### 2.4.3 Na+/K+-ATPase Activity

The reaction mixture for Na^+^/K^+^-ATPase activity measurement included S1, 3 mM MgCl_2_, 125 mM NaCl, 20 mM KCl, and 50 mM Tris/HCl (pH 7.4), with a total volume of 500 μL. Upon the addition of adenosine triphosphate (ATP) to a final concentration of 3 mM, the reaction was initiated. Control assays were performed identically with an additional 1 mM ouabain. The activity of Na^+^/K^+^-ATPase was assessed by calculating the difference in the inorganic phosphate (Pi) released between the two tests. The data were expressed as nmol Pi per mg of protein.^24^

#### 2.4.4 Markers of General Toxicity

Whole blood was centrifuged at 3000 rpm for 10 min to obtain plasma. This plasma was then used to assess the activities of aspartate aminotransferase (AST) and alanine aminotransferase (ALT), as well as creatinine levels, using commercial kits (Bioclin-K048, K049 and K222, respectively). For the analysis of AST activity, 100 μL of plasma and 1.0 mL of the working reagent (Tris buffer < 200 mmol/L, sodium aspartate < 450 mmol/L, d-lactate dehydrogenase 5 KU, malate dehydrogenase 5 KU, chelating agent, alpha-ketoglutaric acid 180 mmol/L, NADH 5 mmol/L, surfactant, and preservative) were used. After 1 min, the mixture was transferred to a thermostated cuvette, and 3 readings were taken at 1 min intervals using a spectrophotometer at 340 nm. The average of the absorbance differences per minute was used for the final calculation. The same procedure was performed for the analysis of ALT activity but using a different working reagent (Tris buffer 200 mmol/L (pH 7.8), LDH 2400 U/L, l-alanine 500 mmol/L, alpha-ketoglutarate 100 mmol/L, NADH 5 mmol/L, and preservative). For the analysis of creatinine levels, 100 μL of the sample was added to 1 mL of the working reagent containing sodium hydroxide 500 mmol/L, sodium carbonate 75 mmol/L, and picric acid 60 mmol/L. After homogenization, the mixture was immediately transferred to a thermostated cuvette at 37 °C, and the absorbance was measured at 510 nm at 30 and 90 s of reaction. The delta absorbance was used to calculate the creatinine concentration. Results were presented as units per liter (U/L) for AST and ALT activities and milligrams per milliliter (mg/mL) for creatinine levels.

#### 2.4.5 Protein Levels

Protein concentration in S1 was evaluated using the Bradford assay, where samples (including bovine serum albumin standards) were diluted 1:50 in TFK buffer (10 mM, pH 7.4) and incubated with Bradford reagent at room temperature for 10 min. Protein levels were detected at 595 nm.^25^

### 2.5 Statistical Analyses

Data are expressed as the mean ± standard error of the mean (SEM). The D’Agostino–Pearson test confirmed a Gaussian distribution. Statistical analysis was carried out using the unpaired Student *t* test. Values of *p* < 0.05 were considered statistically significant. All statistical analyses were conducted using the GraphPad 8 software. Outliers were identified and excluded using the ROUT test for extreme values.

## 3 Results

### 3.1 Open Field Test

**Figure 2:**
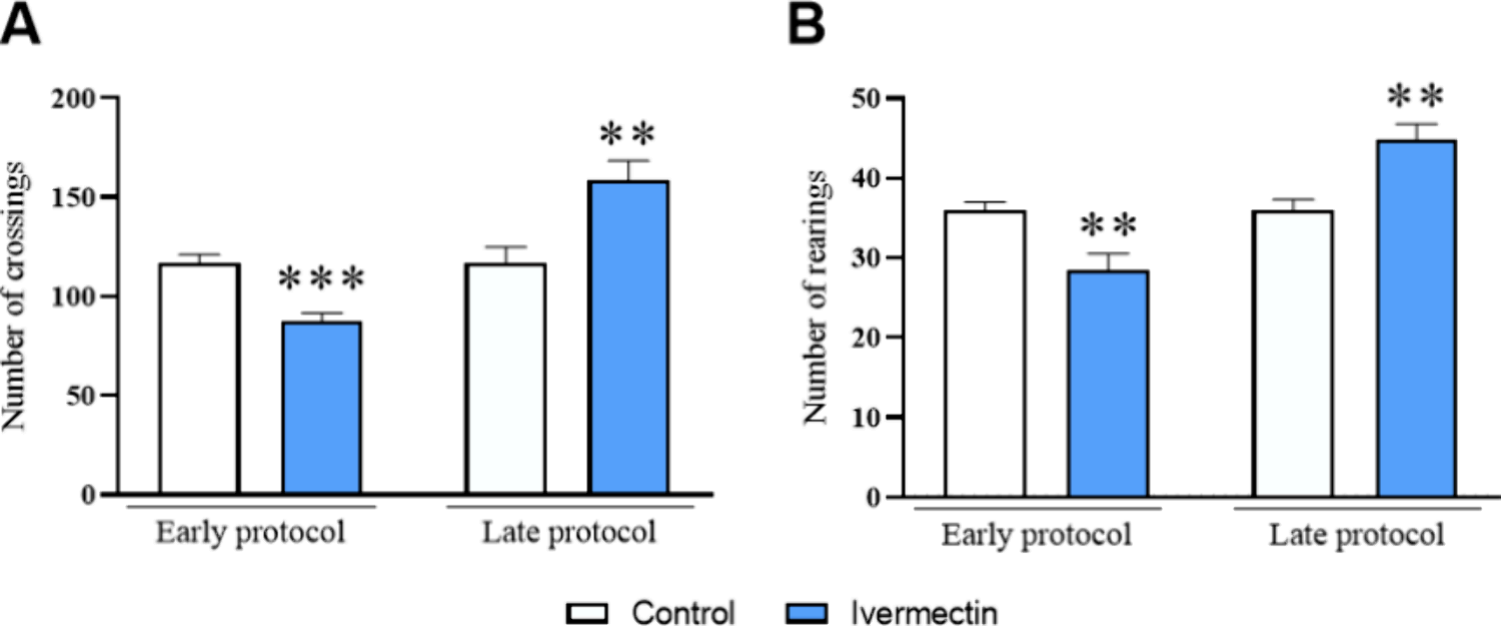
Effects of ivermectin treatment on rat locomotor function in the open field test. (A) Number of crossings and (B) number of rearings in the open field test. Data are expressed as mean ± SEM (*n* = 10 animals per group). The data were analyzed using the unpaired Student *t* test. **p < 0.01 and ***p < 0.001 indicate a significant difference compared to the control group.

Significant differences in motor activity were observed between the control and the ivermectin groups. In the early protocol, the ivermectin group exhibited a decrease in the number of crossings (*p* = 0.0001, *t* = 4.900), followed by an increase in the late protocol (*p* = 0.0039, *t* = 3.312) (Figure 2A). Similarly, the number of rearings decreased in the early protocol (*p* = 0.0043, *t* = 3.266) and increased in the late protocol (*p* = 0.0013, *t* = 3.797) in the ivermectin group (Figure 2B).

### 3.2 Elevated Plus Maze

**Figure 3:**
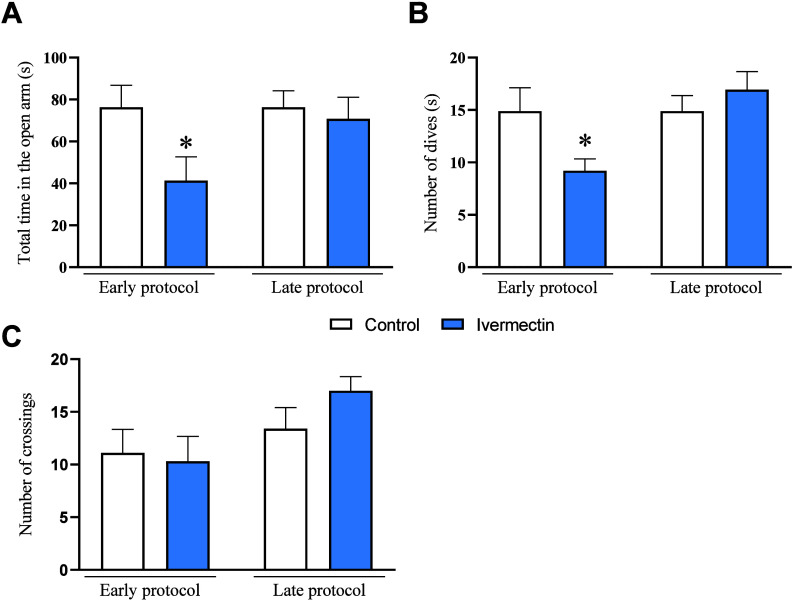
Effects of ivermectin treatment on rat anxiety-like behavior in the elevated plus maze test. (A) Total time in the open arm; (B) number of dives; (C) number of crossings performed by the rats. Data were expressed as mean ± SEM, with 10 animals per group. The data were analyzed using the unpaired Student *t* test. *p < 0.05 indicate a significant difference compared to the control group.

The results from the EPM (Figure 3A) indicated that rats exposed to ivermectin in the early protocol spent less time in the open arms (*p* = 0.0354; *t* = 2.274), indicating anxious-like behavior. However, 13 days after the last ivermectin administration (late protocol), no significant difference was observed (*p* = 0.6723; *t* = 0.4300). Similarly, the number of dives (Figure 3B) significantly diminished in the ivermectin group compared to that in the control group during the early protocol (*p* = 0.0365; *t* = 2.260), although no significant differences were noted in the late protocol (*p* = 0.3808; *t* = 0.8985). Regarding the number of crossings (Figure 3C), neither the early (*p* = 0.8090; *t* = 0.2453) nor the late (*p* = 0.1539; *t* = 1.489) protocols showed significant differences.

### 3.3 Splash Test

**Figure 4:**
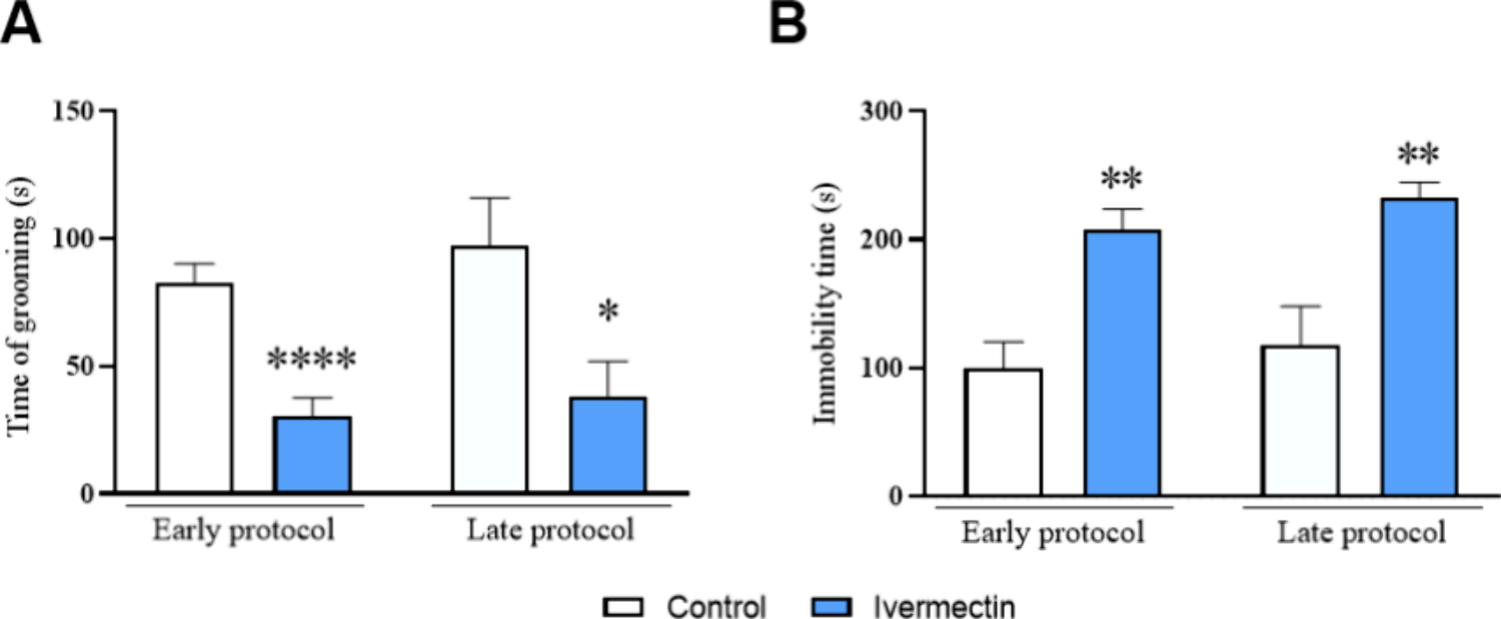
Effects of ivermectin treatment on rat anhedonic and depressive-like behavior observed in the splash test and forced swim test. (A) Time of grooming in the splash test and (B) immobility time in the forced swim test. The data are expressed as mean ± SEM, with *n* = 10 animals per group (Figure A); 6 animals per group (Figure B). Analysis was conducted using the unpaired Student *t* test. *p < 0.05; **p < 0.01 and ****p < 0.0001 indicate a significant difference compared to the control group.

The ivermectin group exhibited a significant reduction in self-grooming time compared with the control group in the early protocol (*p* = 0.0001; *t* = 5.032). This result persisted in the late protocol (*p* = 0.0203; *t* = 2.545), suggesting that the anhedonic effect induced by ivermectin persists for days after treatment cessation (Figure 4A).

### 3.4 Forced Swim Test

Analysis of FST data revealed a significant increase in the immobility time in the ivermectin group in both the early (*p* = 0.0018; *t* = 4.196) and late (*p* = 0.0052; *t* = 3.553) protocols (Figure 4B). Thus, the depressive-like behavior induced by ivermectin persists for days.

### 3.5 Acetylcholinesterase and Na^+^K^+^-ATPase Activities

**Figure 5:**
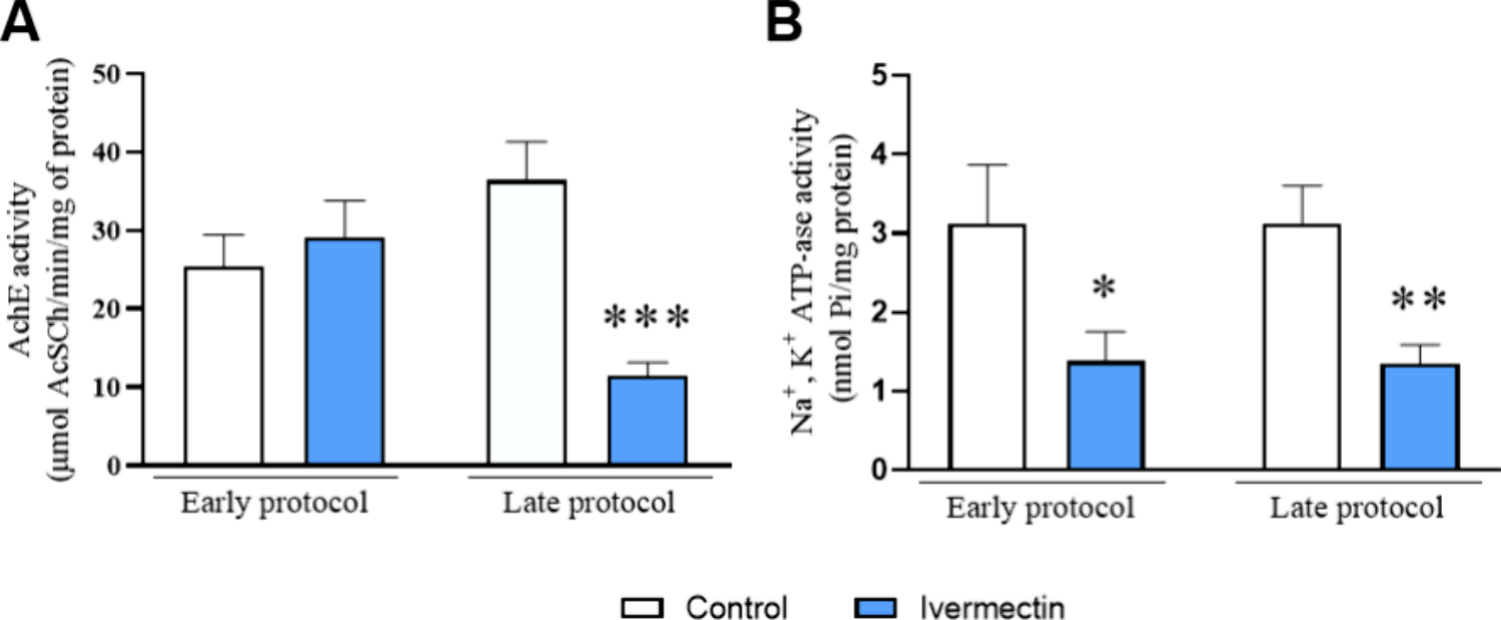
Effects of ivermectin treatment on (A) AChE and (B) Na^+^, K^+^-ATPase activities in the prefrontal cortex of rats are presented. Values are expressed as the mean ± SEM, with *n* = 8–10 animals per group (Figure A); 10 animals per group (Figure B). Data were analyzed using the unpaired Student *t* test. *p < 0.05, **p < 0.01, and ***p < 0.001 indicate a significant difference compared to the control group.

No difference was observed in the AChE activity in the prefrontal cortex between the control and ivermectin groups in the early protocol (*p* = 0.5752; *t* = 0.5721). However, inhibition of AChE activity was noted in the prefrontal cortices of the ivermectin group 14 days after the last drug administration (*p* = 0.0003; *t* = 4.570) (Figure 5A). Regarding Na^+^K^+^-ATPase activity, a significant inhibition in the ivermectin groups was observed in both the early (*p* = 0.0494; *t* = 2.126) and late protocols (*p* = 0.0039; *t* = 3.367) (Figure 5B).

### 3.6 Oxidative Stress Parameters

**Figure 6:**
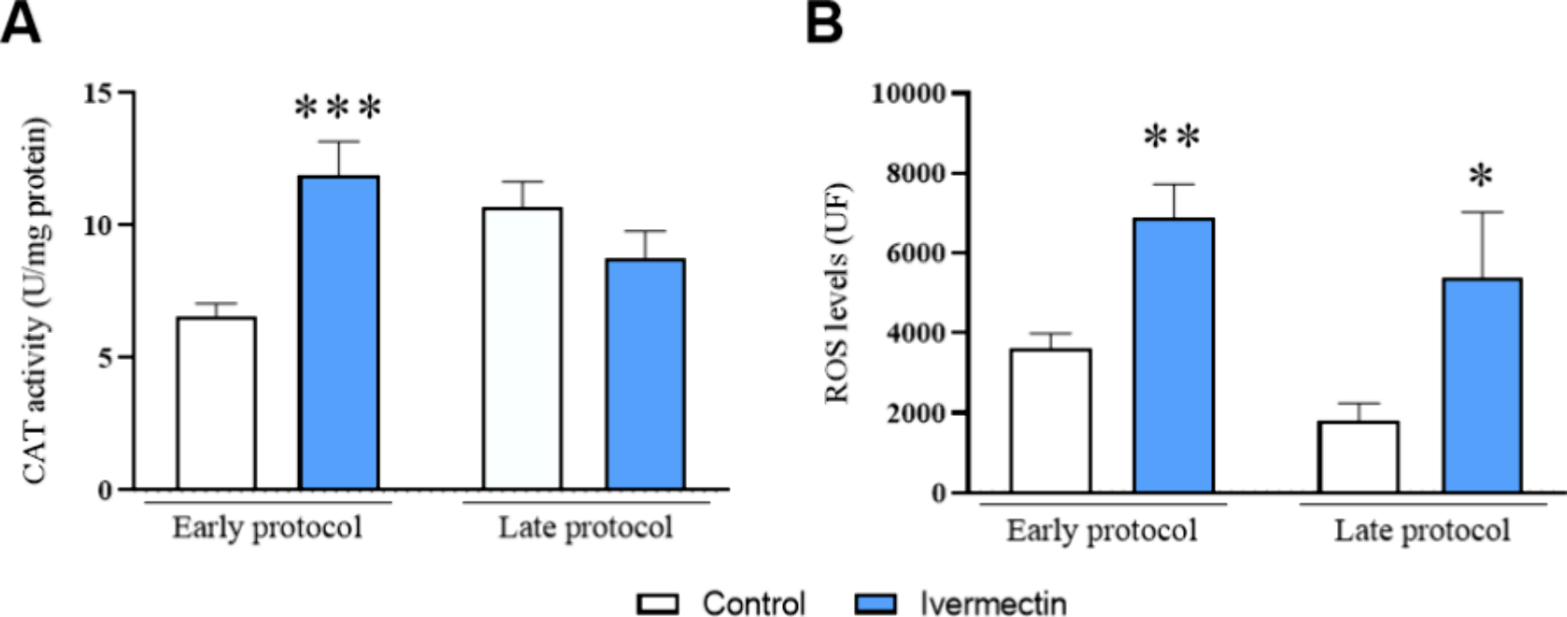
Effects of ivermectin treatment on (A) CAT activity and (B) ROS levels in the prefrontal cortex of rats. The values are presented as the mean ± SEM, with *n* = 8–10 animals per group (Figure A); 10 animals per group (Figure B). The data were analyzed using the unpaired Student *t* test. *p < 0.05, **p < 0.01, and ***p < 0.001 indicate a significant difference compared to the control group.

Oral administration of ivermectin enhanced the CAT activity in the prefrontal cortex of rats 24 h after the last administration of ivermectin (*p* = 0.0006; *t* = 4.229). However, no significant difference was observed between the ivermectin and control groups 14 days later (*p* = 0.1874; *t* = 1.370) (Figure 6A). Regarding the levels of ROS, data analysis demonstrated that ivermectin administration increased ROS levels in the prefrontal cortex of rats in both the early and late protocols (*p* = 0.0022; *t* = 3.634) and (*p* = 0.0498; *t* = 2.122), respectively (Figure 6B).

### 3.7 Markers of General Toxicity

The results presented in Table 1 indicate liver and kidney damage as well as changes in body weight during the experimental protocols. A marked increase in AST levels was noted in the ivermectin group at both 24 h (*p* = 0.0103; *t* = 2.867) and 14 days (*p* = 0.0125; *t* = 2.793) postadministration, compared to the control group. The ALT levels also showed a significant increase at both time points: 24 h (*p* = 0.0002; *t* = 4.657) and 14 days (*p* = 0.0034; *t* = 3.374) after ivermectin administration. Additionally, creatinine levels were significantly elevated in the ivermectin group during the early protocol (*p* = 0.0039; *t* = 3.313). However, no significant difference was observed between the ivermectin and control groups 14 days later (*p* = 0.2081; *t* = 1.306). Moreover, the ivermectin-treated group exhibited a decrease in weight gain during the 5 day administration period (*p* = 0.0060; *t* = 3.044), along with reduced weight gain compared to the control group at the end of the late protocol (*p* = 0.0440; *t* = 2.166) (Table 1).

**Table 1:** Weight Gain and Biochemical Markers of Nephrotoxicity and Hepatotoxicity in the Plasma of Rats Exposed to Ivermectin^a^

	**early protocol**		**late protocol**	
**variables**	control	ivermectin	control	ivermectin
AST (U/L)	74.07 ± 7.51	108.8 ± 9.51*	81.4 ± 7.39	129.8 ± 16.36*
ALT (U/L)	28.23 ± 1.81	38.06 ± 1.09***	24.79 ± 1.01	34.32 ± 2.64**
creatinine (mg/dL)	0.337 ± 0.024	0.515 ± 0.048**	0.377 ± 0.036	0.458 ± 0.051
weight gain (g)	8.083 ± 2.379	–1.417 ± 2.021**	39.9 ± 4.378	26.2 ± 4.565*

a Data were expressed as mean ± SEM (*n* = 9 animals per group for plasma analyses and 12 animals per group for Weight gain analyses). The data were analyzed using the unpaired Student *t* test. Abbreviations: AST: aspartato aminotransferase; ALT: alanina aminotransferase. *p < 0.05, **p < 0.01, and ***p < 0.001 indicate a significant difference compared to the control group.

## 4 Discussion

The present study aimed to investigate the behavioral and physiological effects of ivermectin treatment in rats, focusing on its potential neurotoxic and systemic implications. Our findings revealed complex and multifaceted alterations induced by ivermectin administration, including changes in locomotor activity, anxiety and depressive-like behaviors, oxidative stress, and dysregulation and toxicity of AChE and Na^+^/K^+^-ATPase activities.

Ivermectin, a 16-membered lactone isolated in 1974, is an antiparasitic medication extensively used for its effectiveness in eliminating various parasites while causing minimal effects on the host. Healthcare professionals regulate and prescribe its use to ensure its safe and effective application.^26^ However, studies have indicated that, even at therapeutic doses, ivermectin can induce neurobehavioral changes^27,28^ or exacerbate behavioral and neurochemical disorders.^29^

Consistent with previous literature, our results demonstrated significant alterations in motor function following ivermectin treatment.^28−30^ Initially, rats exhibited decreased motor activity, followed by a rebound increase in activity. This biphasic response may reflect the acute inhibitory effects of ivermectin on central nervous system function, followed by compensatory mechanisms or delayed neuroadaptive changes. The reduced locomotion in rats exposed to acute and early protocols may be attributed to the sedative effects of high doses of ivermectin, which induces depression of the central and peripheral nervous systems.^31^

Ivermectin modulates the GABAergic system by binding to allosteric sites on GABA_A_ receptors, enhancing the receptor’s affinity for GABA, which leads to an increased influx of chloride ions into neurons.^32,33^ The resulting hyperpolarization inhibits action potential firing, reducing neuronal excitability. Consequently, ivermectin exerts sedative effects^31^ and, at lower doses, may exhibit anxiolytic properties.^29,30^ This pronounced sedation could obscure the detection of anxious or depressant-like behavior, as evidenced in the EPM, splash test, and FST in the early protocol. The hypolocomotion and anxiety-like behavior observed in the OFT and EPM disappeared over time, as they were not observed in the late protocol.

Thus, although a reduction in the time spent in the open arms of the EPM, a decrease in grooming in the splash test, and increased immobility in the FST were observed in the early protocol, suggesting potential alterations in anxious and depressive behavior, we postulate that the sedative effects of ivermectin may have interfered with the accurate detection of these behaviors. The sedative properties of ivermectin likely reduced locomotor activity, which could have masked any anxiolytic effects and led to an overestimation of anxiety-like and depressive-like behaviors. Therefore, we did not conclude that ivermectin induced anxiety-like and depressive-like alterations in behavior during the early protocol. In contrast, during the late protocol, as the sedative effects of the drug dissipated, it is possible to conclude that the rats exhibited depressive-like behavior.

Alterations were observed in the FST and splash test in the late protocol, indicating a persisting depression-like effect induced by ivermectin for at least 14 days. The anhedonic and depressive-like behaviors in rats treated with ivermectin were evidenced by an increased immobility time in the FST and decreased grooming time in the splash test. During this period, no hypolocomotion was observed; instead, the rats displayed an increased locomotor activity. Bortolato^34^ reported that low doses of ivermectin resulted in heightened depression-related markers in both the tail suspension test and FST without affecting the distance traveled in the open field or inducing toxicity.

Previous research by Basudde^35^ demonstrated that high doses of ivermectin could induce depressive symptoms in animals. His study showed that administering ivermectin to calves increased serum pseudocholinesterase levels, indicating an impact on the cholinergic nervous system associated with GABA-mediated cholinergic function. This study observed a delayed inhibition of AChE following exposure to ivermectin, which may be at least partially related to the depressive-like effects induced by this drug in the late protocol. Recent studies have shown that AChE inhibitors, including drugs prescribed for dementia or organophosphate compounds, may be linked to increased depression.^36−38^

Additionally, we hypothesize that the AChE inhibition observed in the late protocol may be related to hyperlocomotion induced by ivermectin. The rise in acetylcholine levels due to AChE inhibition could contribute to a more hyperactive behavioral phenotype, as evidenced by an increased number of crossings and rearing behaviors.^29,39,40^ Parisi et al. also demonstrated that ivermectin induces hyperlocomotion in juvenile rats.^29^

Another significant finding was that ivermectin induced Na^+^, K^+^-ATPase inhibition in the prefrontal cortex of rats in both early and late protocols. These results suggest that the combined inhibition of Na^+^, K^+^-ATPase, and AChE may have a synergistic effect on neural function, leading to the observed behavioral changes. Na^+^, K^+^-ATPase is essential for maintaining ionic balance and synaptic transmission,^41^ and its downregulation can lead to electrochemical imbalances in synaptic transmissions, negatively affecting behavioral functions such as locomotion. Similarly, Serafini et al.^42^ exposed fish to different high concentrations of eprinomectin (a member of the avermectin class), leading to the inhibition of both enzymes in the later protocol and likely causing more severe disruption in neural activity. This accounts for the observed hyperlocomotion, highlighting the crucial roles that these enzymes play in regulating synaptic function and behavior.

A correlation exists between decreased Na^+^, K^+^-ATPase activity in the brain and increased production of ROS. The excessive production of ROS may impede Na^+^, K^+^-ATPase function, a sulfhydryl enzyme sensitive to oxidative stress. This, in turn, could influence the behavioral changes observed in the study.^42^ Furthermore, the study’s results indicated elevated ROS levels and disrupted CAT activity, suggesting oxidative stress might follow ivermectin treatment. Consistent with previous findings, exposure to ivermectin has been associated with oxidative stress in cellular systems, as indicated by an increase in ROS levels, leading to DNA damage.^43^

Oxidative stress results from an imbalance between ROS and the defense system meant to neutralize these ROS, preventing damage.^44^ Excess ROS can cause tissue damage and are associated with neurobehavioral problems such as depression.^45^ Additionally, CAT, an important antioxidant enzyme, helps scavenge hydrogen peroxide, a major ROS, reducing the level of oxidative cell damage. The increase in CAT activity may serve as a compensatory mechanism to counteract the enhanced ROS production induced by ivermectin.^46^ However, an increase in CAT activity was not observed in the late protocol. However, since ROS levels remained high, it is likely that other antioxidant enzymes were involved in this attempt to maintain redox balance. Studies have shown that H_2_O_2_ acts as a “suicide substrate” at high concentrations (>100 μM), leading to the irreversible inactivation of catalase.^47,48^ In this sense, it is plausible that the elevated ROS levels themselves may have decreased the previously increased CAT activity. Under these conditions, H_2_O_2_ is detoxified primarily by GPx.

For future studies, we highlight limitations and perspectives that could enhance our understanding of the effects of high doses of ivermectin. First, while we assessed oxidative stress through CAT activity and ROS levels, future research could build on this by incorporating additional markers of oxidative stress, such as antioxidant enzymes and indicators of lipid or protein damage, for a more comprehensive assessment. Moreover, measuring neurotransmitter levels, including serotonin, GABA, dopamine, and glutamate, would offer valuable insights into the neurobiological mechanisms underlying depression and anxiety, further elucidating the behavioral changes observed.

In addition to these considerations, following OECD guidelines for acute toxicity studies,^49^ the protocol involved using high doses of ivermectin on female Wistar rats. The general toxicity analysis showed hepatotoxic effects from ivermectin administration in the early protocol, which persisted in the late protocol. The literature review revealed cases of liver damage, such as a woman who, after a single dose of ivermectin for a parasitic infection, died 30 days later from severe hepatitis, confirmed by biopsy to be drug-induced.^50^

In conclusion, this study investigated the impact of high-dose ivermectin treatment in rats, shedding light on its neurotoxic and systemic implications. The findings indicate that ivermectin leads to significant changes in locomotor activity, depressive-like behavior, oxidative stress, and dysregulation of AChE and Na+ and K+-ATPase activities. The biphasic response in motor function, coupled with persistent depression-like effects and enzyme inhibition, suggests a complex interaction between ivermectin and neural function. The study also highlighted the role of oxidative stress in mediating these effects, as evidenced by elevated ROS levels and disrupted CAT activity. Future research should aim to explore the effects of nontoxic doses of ivermectin on neurobehavior to further understand its safety profile.
